# Identification of *Anaplasma marginale* Type IV Secretion System Effector Proteins

**DOI:** 10.1371/journal.pone.0027724

**Published:** 2011-11-28

**Authors:** Svetlana Lockwood, Daniel E. Voth, Kelly A. Brayton, Paul A. Beare, Wendy C. Brown, Robert A. Heinzen, Shira L. Broschat

**Affiliations:** 1 School of Electrical Engineering and Computer Science, Washington State University, Pullman, Washington, United States of America; 2 Department of Microbiology and Immunology, University of Arkansas for Medical Sciences, Little Rock, Arkansas, United States of America; 3 Department of Veterinary Microbiology and Pathology and Paul G. Allen School for Global Animal Health, Washington State University, Pullman, Washington, United States of America; 4 Coxiella Pathogenesis Section, Laboratory of Intracellular Parasites, Rocky Mountain Laboratories, National Institute of Allergy and Infectious Diseases, National Institutes of Health, Hamilton, Montana, United States of America; University of Minnesota, United States of America

## Abstract

**Background:**

*Anaplasma marginale*, an obligate intracellular alphaproteobacterium in the order Rickettsiales, is a tick-borne pathogen and the leading cause of anaplasmosis in cattle worldwide. Complete genome sequencing of *A. marginale* revealed that it has a type IV secretion system (T4SS). The T4SS is one of seven known types of secretion systems utilized by bacteria, with the type III and IV secretion systems particularly prevalent among pathogenic Gram-negative bacteria. The T4SS is predicted to play an important role in the invasion and pathogenesis of *A. marginale* by translocating effector proteins across its membrane into eukaryotic target cells. However, T4SS effector proteins have not been identified and tested in the laboratory until now.

**Results:**

By combining computational methods with phylogenetic analysis and sequence identity searches, we identified a subset of potential T4SS effectors in *A. marginale* strain St. Maries and chose six for laboratory testing. Four (AM185, AM470, AM705 [AnkA], and AM1141) of these six proteins were translocated in a T4SS-dependent manner using *Legionella pneumophila* as a reporter system.

**Conclusions:**

The algorithm employed to find T4SS effector proteins in *A. marginale* identified four such proteins that were verified by laboratory testing. *L. pneumophila* was shown to work as a model system for *A. marginale* and thus can be used as a screening tool for *A. marginale* effector proteins. The first T4SS effector proteins for *A. marginale* have been identified in this work.

## Introduction

The type IV secretion system (T4SS) is found in a diverse set of microorganisms—including both Gram-negative and Gram-positive bacteria—that infect a variety of animal and plant hosts. While the core genes of the T4SS are somewhat conserved among organisms, the complement, gene order, number of homologs, and sequence composition vary greatly from organism to organism [1], [2].

Members of the order Rickettsiales comprise several animal and human pathogens. Systematic studies of the genomes of these organisms have revealed the presence of the T4SS [3], [4]. The T4SS of Rickettsiales is characterized by an expansion of the *virB4* and *virB6* gene families and an absence of *virB5*; in addition, species in the family Anaplasmataceae have an expansion of *virB2* and are missing *virB1* [5]. The identification of T4SSs naturally prompted a search for effector molecules secreted by these systems in order to identify mechanisms of virulence and pathogenesis. However, discovery of effector proteins in Anaplasmataceae is hampered by the lack of both reliable prediction algorithms and systems for genetic modification. For most microorganisms with T4SSs only a handful of effectors are known [1] with the exception of *Coxiella burnetii* with 60 effector proteins [6], [7], [8] and *Legionella pneumophila* with 145 effector proteins [9].

The rickettsial pathogen *Anaplasma marginale*, a member of the family Anaplasmataceae, is an obligate, intracellular tick-borne pathogen that causes anaplasmosis in cattle. The T4SS in *A. marginale* is thought to play an important role in invasion and pathogenesis by translocating effector proteins across the pathogen membrane into eukaryotic target cells. To facilitate the study of effector proteins in *A. marginale*, an algorithm for T4SS effector prediction is needed. However, development of accurate machine learning prediction algorithms requires sets of known negative and positive effector proteins. In the absence of these data for *A. marginale*, we developed an approach to identify a set of effector proteins that combined computational methods with functional testing using the *L. pneumophila* reporter system. *L. pneumophila* has been previously used to validate secretion of *C. burnetti* and *A. phagocytophilum* [10] effectors and is predicted to be similar to the rickettsial T4SS by several classification systems [5], [11]. This study provides the first report of secreted effector proteins for *A. marginale* and validates the use of *L. pneumophila* as a system to test effector secretion for rickettsial pathogens. The results obtained afford a step toward the goal of developing a machine learning algorithm that will provide a robust means of predicting effector proteins.

## Results

### Identification of potential effector proteins

After comparing the properties of known T4SS effector proteins with the properties of *A. marginale* housekeeping genes (Tables S1, S2, S3, and S4), the following procedure was applied to select a subset of potential effector proteins. First, we selected a hydropathy cutoff value. To do this, we looked at the hydropathy values for known T4SS effectors. In particular, none of the known effectors of *Bartonella henselae* (Table S3) has a hydropathy value greater than −265. Among *A. tumefaciens* effectors one has a hydropathy value of −112, but the average of the remaining four effectors is −529.8 (Table S2). The most abundant set of T4SS effectors is known for *L. pneumophila*; these effector proteins have a more diverse array of hydropathy values, with an average hydropathy of −261 and median value of −211 (Table S4). Based on these observations, *A. marginale* proteins were filtered leaving only those whose total hydropathy score was less than −200, as this condition selects proteins with strong hydrophilic profiles. Next, we selected only proteins with hydrophilic tails, i.e., those for which 25 amino acids at the C-terminus have a combined negative hydropathy. Third, proteins with known housekeeping functions and/or with predicted localization signals (i.e. signal peptides) were removed from consideration [12]. The resulting 21 proteins were ordered with higher ranking given to proteins with strong negative average hydropathy (Table 1). Although the results of sequence identity searches against known effector proteins were not strong, proteins that showed some level of similarity to known T4SS effectors were preferentially selected for laboratory testing. Additionally, two other factors were considered. First, proteins with a “eukaryotic domain” were considered to be likely effectors because bacterial proteins bearing such domains potentially mimic eukaryotic host cell functionality. In particular, proteins bearing ankyrin repeat domains (ANKs) have a high probability of being T4SS effector proteins [7], [13]. The second factor was whether previous data indicated that a particular gene was up regulated in tick cell culture [14]. *A. marginale* transits between erythrocytes and tick cells, and it is expected that effector proteins are more likely to play a role in the biology of nucleated tick cells.

**Table 1:** Predicted *A. marginale* effector proteins.

Locus ID	Length^1^	Hydro^2^	C-term charge^3^	C-term hydro^4^	Avg Hydro^5^	Protein Description	Euk Domain^6^	Seq ID^7^	Tick^8^
AM1141	367	−302.3	+7	−52.3	−0.82	hypothetical		BH	X
AM185	798	−583.6	+4	−35	−0.73	hypothetical	CC	LP (1)	
AM470	1261	−804	+4	−13.3	−0.64	hypothetical			X
AM742	458	−270.7	−8	−24.5	−0.59	hypothetical			
AM410	416	−218.4	+1	−3.9	−0.53	hypothetical		AP: Ats-1	X
AM638	3194	−1689.8	+1	−42.2	−0.53	AnkB	ANK, CC	LP (4)	
AM354	899	−468.5	−2	−5.4	−0.52	hypothetical			
AM347	1200	−580.5	+3	−33.5	−0.48	hypothetical			
AM366	2839	−1328.9	+3	−23.9	−0.47	hypothetical			
AM356	1536	−711.3	+3	−9	−0.46	hypothetical			
AM034	813	−358.4	−2	−29	−0.44	hypothetical			
AM071	1329	−576.7	+2	−15.1	−0.43	hypothetical		LP (2)	
AM810	1687	−710.3	−3	−14.8	−0.42	hypothetical	CC		
AM613	506	−203.9	−1	−21.3	−0.4	hypothetical			X
AM705	1387	−552.8	+3	−24.9	−0.4	AnkA	ANK	AP: AnkA, LP (3)	
AM072	2039	−750.9	0	−1.1	−0.37	hypothetical			
AM387	1486	−510.5	+5	−22.6	−0.34	hypothetical		LP (2)	
AM402	880	−292.9	+7	−13.2	−0.33	hypothetical			
AM689	1014	−260	+3	−22.5	−0.26	hypothetical			
AM540	2513	−546	+2	−22.9	−0.22	hypothetical			
AM712	3492	−725.3	−2	−19.4	−0.21	hypothetical		LP (5)	

1 Protein length in amino acids.

2 Hydropathy of total protein.

3 Charge of C-terminal 25 amino acids.

4 Hydropathy of C-terminal 25 amino acids.

5 Average hydropathy = total hydropathy/length.

6 Presence of eukaryotic domain: CC = coiled coil domain, ANK = ankyrin repeat domain.

7 Has sequence identity to a known effector molecule. Effector known in AP = *A. phagocytophilum*, LP = *L. pneumophila* (proteins marked in last column of Table S4), BH = 20 aa's on C-terminus of BepD (UniProt ID Q6G2A5) in *B. henselae*. Number in parenthesis indicates the number of hits when BLASTed against the effector pool.

8 Refers to genes shown to be transcribed in tick cell culture.

**Figure 1:**
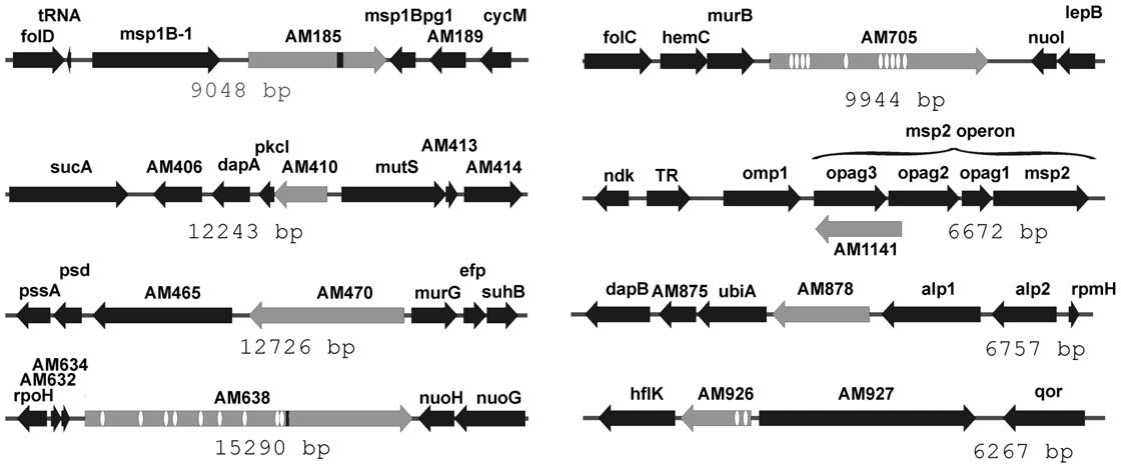
Genomic arrangement of genes tested in this study. The gene of interest is highlighted in gray. Beginning positions for each indicated region are: AM185 - 152987, AM410 - 361688, AM470 - 416086, AM638 (*ankC*) - 570776, AM705 (*ankA*) - 636928, AM1141 - 1025923, AM878 (*aaap*) - 806001, AM926 (*ankB*) - 843819. Numbers (bp) under each locus indicate the length of each depicted region. The white ovals indicate positions of ankyrin repeats, and black bars indicate the coiled coil domains.

Six proteins, AM185, AM410, AM470, AM638, AM705, and AM1141, were chosen for functional testing. Each of these proteins has been detected in previous proteomic studies, suggesting that while they are proteins of unknown function, they are, in fact, synthesized in *A. marginale* [14], [15], [16]. AM705 contains ANK domains and is a predicted homolog of *A. phagocytophilum* AnkA, a protein that is translocated to the nucleus in a T4SS-dependent manner [15], [17], [18]. AM638 (AnkC) also contains ANK domains. AM1141 is notable in that it is encoded on the opposite strand to the *msp2* operon, a well studied operon that is transcribed in all life stages of *A. marginale* [19]. It is unusual for genes to be transcribed from both strands, and interestingly, the Opag3 protein appears to be absent in tick cells where AM1141 has been detected [14], [19]. In addition, two proteins were chosen for functional testing as a type of negative control; translocation of one or both of these proteins would demonstrate that the algorithm failed to predict a T4SS effector. It is not actually possible to choose a true negative control because lack of translocation does not mean a protein is not a T4SS effector; only the converse is true. The two proteins selected were AM878, encoding *Anaplasma* appendage associated protein (Aaap), chosen because it is known to be secreted and does not contain a signal peptide, and AM926 (AnkB), a third ANK domain containing protein [15], [20]. Both proteins have at least one hallmark of an effector but do not meet the hydropathy criteria of our algorithm and thus were not predicted to be effectors. The genomic arrangement of the genes encoding these proteins with their predicted eukaryotic domains is shown in Figure 1.

### Experimental validation of effector proteins

**Figure 2:**
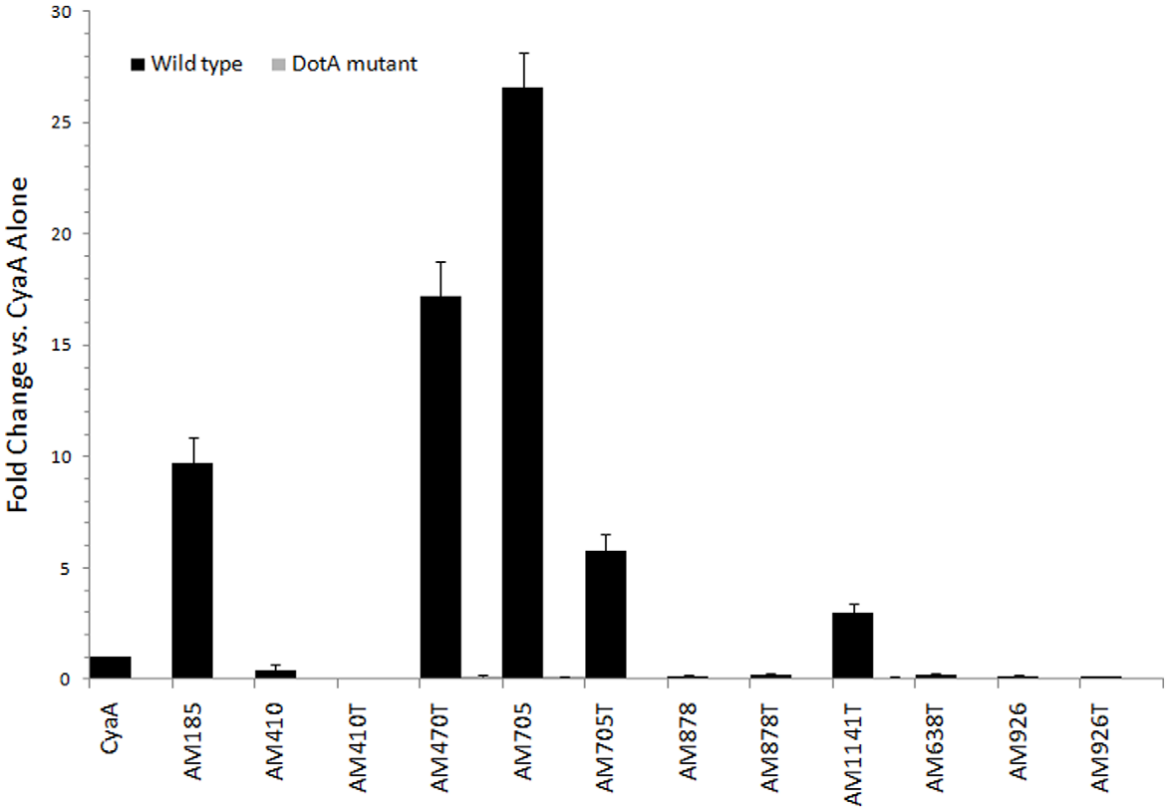
CyaA translocation assays. Intracellular cAMP levels were determined following infection of THP-1 cells with *L. pneumophila* expressing CyaA fused to individual *A. marginale* proteins. Results are expressed as fold change over cAMP levels resulting from infection with *L. pneumophila* expressing CyaA alone (negative control). Increased cAMP levels were observed when AM185, AM470T, AM705 (*ankA*), AM705T, and AM1141T fusion proteins were expressed in wild-type *L. pneumophila*, and levels similar to the negative-control were observed following expression of all proteins in DotA-deficient *L. pneumophila*, indicating that secretion requires a functional Dot/Icm T4SS. Results are shown for one experiment performed in triplicate and are representative of at least 2 individual experiments. Error bars represent the standard deviation from the mean. *p* values are <0.0001 using a Student's t-test when comparing secreted AM proteins to CyaA alone or to the DotA mutant cAMP levels.

Each of the candidate genes was cloned as a full length gene or a truncation encoding the C-terminal 100 amino acids fused to *Bordetella pertussis* adenylate cyclase (CyaA) gene, with the exception of AM638, which was only cloned in the truncated form as the full length protein is extremely large (3194 amino acids). Fusion constructs were tested for secretion by the *Legionella pneumophila* Dot/Icm T4SS that has successfully identified *C. burnetii* and *A. phagocytophilum* T4SS effector proteins [7], [10], [21]. Truncated constructs were generated because the Dot/Icm system of *L. pneumophila* recognizes C-terminal translocation signals within effector proteins [7], [22]. All proteins were expressed at the correct size in *L. pneumophila* (Fig. S1), with the exception of full length AM1141 (data not shown). THP-1 human macrophage-like cells were infected with *L. pneumophila* transformants harboring *cyaA* fusion plasmids and assayed for elevated cAMP levels. The CyaA assay depends on the translocation signal within the *A. marginale* portion of the fusion protein to deliver CyaA to the cytosol of the host cell, where CyaA is activated by calmodulin to produce cAMP [23]. The results shown in Figure 2 reveal that four of the six *A. marginale* predicted effector proteins directed CyaA translocation to the host cytosol when either the full length protein (AM185), truncation mutant (AM1141, AM470), or both (AM705) were expressed in *L. pneumophila*. The two proteins that were not predicted to be translocated in a T4SS-dependent manner did not direct CyaA to the host cytosol. To confirm that translocation was Dot/Icm-dependent, proteins that successfully directed translocation were tested using DotA^−^ *L. pneumophila*-infected cells in the CyaA assay. In all cases, use of the DotA^−^ mutant abrogated significant accumulation of cAMP in THP-1 cells (Figure 2). Levels of cAMP in this assay are similar to those shown for *Coxiella burnetii* and *Anaplasma phagocytophilum* effector proteins using the same methods [7], [10].

## Discussion

The study presented here is the first to successfully predict effector proteins for *A. marginale* and demonstrate their translocation in a T4SS-dependent manner. Our goal is to develop an algorithm for predicting T4SS effector proteins; however, this goal is complicated by the fact that very few effector proteins are known for the Anaplasmataceae for use as a training set in machine learning algorithms. Therefore, to select proteins for functional screening as T4SS substrates, we developed a selection scheme based on important features of known T4SS effectors from *Legionella*, *Bartonella*, and *Agrobacterium*. This scheme included measurements of hydropathy for the whole protein and the C-terminal 25 amino acids of the protein and published features suggestive of proteins with a high probability of being an effector. Our selection scheme yielded 21 potential effector proteins. The selection criteria used to identify these proteins are discussed below.

### Hydropathy

Analysis of known effectors from *A. tumefaciens*, *L. pneumophila*, and *B. henselae* revealed that most are hydrophilic in nature, having total negative hydropathy scores, negative average hydropathies, and highly hydrophilic C-termini (Tables S2, S3, S4). As the translocation signal is contained in the C-terminal region of the protein, the hydropathy of this region is of particular interest. These criteria were used in an initial screening of the *A. marginale* proteome and resulted in selection of 33 proteins with hydropathy scores of less than −200, negative average hydropathies, and negative hydropathies at the C-termini.

### “Functional” screening

The list of 33 proteins with hydrophilic characteristics was screened for sequences with known functions, such as housekeeping proteins and surface proteins, and these were removed. Proteins of unknown function containing signal peptides were also removed from the list because the signal peptide would target a protein for secretion through the Type II secretion system [24]. The final list contained 21 candidate proteins (Table 1).

### Eukaryotic domains

An important feature considered was the presence of encoded eukaryotic domains in the *A. marginale* genome. To subvert host cellular processes, pathogens often “hijack” eukaryotic domains to mimic host protein functionality [25], [26], [27]. Thus, we surmised that prokaryotic proteins with eukaryotic features are more likely to be effector proteins. In addition, we analyzed the distribution of encoded eukaryotic domains in *L. pneumophila* strain Philadelphia and found that domains with a high representation ratio in Eukaryota are prevalent among *L. pneumophila* effectors (data not shown). For our study, we defined a eukaryotic domain as a domain that is twice as likely to be present in eukaryotic proteins as in prokaryotic proteins when the SMART domain server is queried. The *A. marginale* St. Maries genome was examined for encoded eukaryotic domains, such as ankyrin repeat domains (Anks), and motifs that facilitate protein-protein interactions, such as coiled coil regions (Table 1). While proteins with such domains were considered to have a higher likelihood of being effector proteins, their absence did not merit exclusion from the set of possible effector proteins.

#### Proteins with ankyrin repeats

Ankyrin (ANK) repeats are domains consisting of 33 amino acids arranged in a helix-turn-helix motif. These structural motifs are the most common protein-protein interaction motif and until recently were thought to be exclusively found in eukaryotic proteins [28], [29]. With the growing availability of a large number of bacterial genome sequences, Anks are becoming increasingly recognized among Proteobacteria and are believed to be acquired via horizontal gene transfer [28], [30]. Proteins bearing ANK motifs are thought to play an important role in microbial pathogenesis by altering host cell function, and a number of ANK-containing proteins are T4SS effectors, including proteins from *L. pneumophila*, *C. burnetti*, and *A. phagocytophilum* [7], [13], [18], [28], [31], [32]. Therefore, proteins containing ANK domains were deemed to be potential T4SS effectors. *A. marginale* has three ANK-domain containing proteins: AnkA, B, and C [15].

#### Proteins with coiled coil domains

Coiled coil domains, structural motifs in which two to five amphipathic α-helices twist together like the strands of a rope, are found in a small subset of proteins ∼2–3% [33]. Coiled coil domains are protein interaction domains with a variety of functions ranging from assembly of macromolecular complexes to molecular recognition. The majority of experimentally verified coiled coil domain containing proteins are eukaryotic in origin [34]. Interestingly, coiled coil domains are prevalent in secreted virulence effector proteins, notably of the type III secretion system [34]. We found 3% of the *A. marginale* proteome to contain predicted coiled coil domains, and three of the 21 proteins identified as potential effectors contain coiled coil domains (Table 1).

#### Other protein domains

Other domains enriched in proteins of eukaryotic origin that were examined included patatin, Miro, Proteasome, UBA, DnaJ, and PDZ; however, these domains did not appear in the final list of proteins with negative average hydropathy.

### Similarity to known effectors

Identification of *A. marginale* effector proteins is complicated by the fact that within Anaplasmataceae only two effector proteins have been experimentally confirmed, AnkA and Ats1 [18], [35]. We identified AM705 (AnkA) and AM410 (Ats1) as homologs of these effectors; however, the percent identity values for these homologs were very low: AnkA, 19% and Ats1, 27%. Among other α-Proteobacteria, *Bartonella henselae* and *A. tumefaciens* have confirmed effector proteins [1]. Although we screened these for sequence identity, we expected to find an even lower degree of identity for these more distantly related organisms and evaluated scores with an e-value as high as 1.2. First, we performed BLAST searches against the entire protein sequence and then only the C-terminal region, which is predicted to contain the translocation signal. The sequence identity search returned insignificant results with the exception of AM1141, which contained low identity to 20 amino acids at the C-terminus of *B. henselae* BepD. We next expanded our search to the γ-Proteobacteria *L. pneumophila* because it has the largest number of experimentally confirmed effector proteins [6], [7]. Six of the 21 *A. marginale* proteins had low similarity scores to one or more effectors from *L. pneumophila* (Table 1). As these sequence identity scores were very low, the lack of a score was insufficient to discount a candidate as an effector, while even low identity was considered when choosing potential effectors for experimental verification.

### Other published data

*A. marginale* transits from the enucleated erythrocyte of the mammalian host to the nucleated cells of the arthropod vector. We reasoned that effectors such as Ats-1, which interferes with apoptosis [35], and AnkA, which traffics to the nucleus and down regulates gene expression [17], are more likely to be up regulated in nucleated cells. Therefore, we included an analysis of *A. marginale* genes/proteins that are up regulated in tick cells [14] (Table 1). The T4SS machinery is translated in the erythrocyte [36], [37], [38], [39] and therefore effectors may also be at work in this environment. The limited dataset of 15 proteins up regulated in tick cell culture was used to enhance available information for selecting candidates.

### Selected proteins for experimental verification

Our selection algorithm is based on qualities of known T4SS effectors in *L. pneumophila*, *Bartonella* spp., and *A. tumefaciens*. The majority of these effector proteins comply with the proposed selection rule, i.e., they have highly hydrophilic profiles both overall and at the C-terminus. However, we must reiterate that our algorithm is an intermediate step toward predicting effector proteins in *A. marginale*. It captures important information for effector proteins, but may also capture some qualities of non-effector proteins resulting in false positives. It may also omit some properties of effector proteins and give rise to false negatives. Given that our selection algorithm is based on qualities of known effector proteins from several different genera, it might be applicable to other organisms for initial identification of T4SS effector proteins. Indeed, the success of the selection method suggests a universal theme for T4SS effectors, including the importance of the hydrophylic profile for the overall length of the protein and its C-terminus, the presence of eukaryotic domains, and the significance of the C-terminus for translocation.

Our results show that four (AM185, AM470, AM705 [AnkA], and AM1141) of the six predicted effectors chosen for experimental verification were translocated in a T4SS-dependent manner by *L. pneumophila*. Importantly, both proteins (AM878 [Aaap] and AM926 [AnkB]) that were not predicted to be T4SS substrates were not translocated in this model. AM705 (AnkA) and AM638 (AnkC) were predicted to be effectors, scoring with low hydropathy and containing ANK domains. Also, AnkA is the homolog of a known effector [15] and AnkC contains a coiled coil domain and low sequence identity with four *L. pneumophila* effectors. AnkA was successfully translocated by *L. pneumophila*, but despite having several hallmarks of effectors, AnkC was not. A distinguishing feature of AnkC is its size; AnkC is the second largest protein encoded by *A. marginale* with a length of 3194 residues. Because of this size, we only tested the C-terminal region of the protein for secretion. Therefore, a separate region of AnkC may be required for efficient recognition and translocation by the T4SS. The third Ank-containing protein, AnkB, was not translocated but also did not meet our criteria and was not predicted to be a candidate by the algorithm. AnkB was chosen for testing because of its ANK domains. Although it has an average hydropathy of −0.56 and a hydrophilic C-terminus, it is relatively small, with a length of 282 residues. This modest length is reflected by the total hydropathy, which is only −158.3. AM410 scored well in the algorithm and was chosen due to Ats-1 homology, but it was not translocated by *L. pneumophila*. AM878 (Aaap) was chosen as a type of negative control as explained previously because it is known to be secreted and does not have a signal peptide [20], and it also was not translocated by our model system. While it is possible that AM410, AM638, AM878, and AM926 are truly non-effectors, it is also possible that *L. pneumophila* is an insufficient reporter system for these proteins; i.e., correct signals may be lacking in the heterologous reporter system. AM1141 was chosen because it has the most hydrophilic C-terminus and is up regulated in tick cell culture. Likewise, AM1141 was successfully translocated by the T4SS. AM1141 is interesting from the standpoint of its genomic location; it resides on the opposite strand from *opag3*, a member of the well studied *msp2* operon [40]. Opag3 is expressed in erythrocytes, but not in tick cells where AM1141 is expressed [14], [19]. This suggests an interesting form of post-transcriptional regulation may occur with these genes. AM185 and AM470 scored well in our selection algorithm and each has a secondary piece of supporting evidence. AM185 has a coiled coil domain and AM470 is up regulated in tick cell culture. While both proteins were translocated by the T4SS, they were translocated only in one form, AM185 as a full length protein and AM470 in the truncated form. This is not unexpected because of differences in folding of the recombinant proteins.

The four translocated proteins are conserved among *A. marginale* sensu stricto strains. AM185 has 99–100% sequence conservation among the strains where it has been fully sequenced (St. Maries, Florida, and Puerto Rico) while AM 470 has 100% sequence identity between the St. Maries and Florida strains (it is not fully present in the high throughput genome sequences that are available, more likely due to technical issues than the absence of the gene in these strains [41]). AM705 (AnkA) ranges from 94–100% sequence identity in the St. Maries, Florida, Mississippi, Puerto Rico, and Virginia strains. Interestingly, we have found less sequence conservation among ANK domain containing proteins, which may be because ANK domains are constrained by structure rather than sequence [15]. Finally, AM1141 presents an interesting case as it is conserved (94–100%) in most *A. marginale* strains examined (St. Maries, Florida, Washington O, Washington Clarkston, South Idaho, and Virginia), but in the Oklahoma strain it has an in-frame stop codon such that protein coding is disrupted. It is perhaps of note that organisms in this order have documented cases of newly arising mutations that lead to “split orfs” such as that observed for the Oklahoma strain; however, the significance of the absence of this protein from the Oklahoma strain is unknown [42], [43]. Importantly, based on our algorithm, all strain variations of these proteins are predicted to be effectors.

The approach employed to predict T4SS effector proteins in *A. marginale* identified four such proteins that were verified by laboratory testing. *L. pneumophila* was shown to function as a model system for *A. marginale* and can now be used as a screening tool for *A. marginale* effector proteins. Importantly, the first T4SS effector proteins for *A. marginale* have been identified in this work.

## Methods

### Sequences

Protein sequences for *Anaplasma marginale* strain St. Maries (NC_004842) and *Legionella pneumophila* strain Philadelphia 1 (NC_002942) were obtained from the NCBI genome database (ftp://ftp.ncbi.nlm.nih.gov/genomes/) in April 2010. Protein sequences for *Bartonella henselae* and *Agrobacterium tumefaciens* (Tables S2, S3) were obtained from UniProtKB (http://www.uniprot.org/) in May 2010.

### Bioinformatics

#### Hydropathy

Hydropathy profiles were calculated using the Kyle-Doolittle scale [44] with each amino acid assigned a positive or negative value depending on hydrophobicity or hydrophilicity, respectively. The charge of a protein was calculated by assigning amino acids H, K, and R values of +1 (positive charge) and E and D values of −1 (negative charge). Average values were calculated based on the total length of residues in a protein.

#### Eukaryotic domains

The *A. marginale* strain St. Maries proteome was scanned for the presence of eukaryotic domains using three independent search engines: NCBI Conserved Domain Search [45], SMART [46], and Pfam [47]. All Web servers were accessed in June 2010. NCBI Conserved Domain Search was used with all default settings. SMART was used in batch mode and included Pfam domains with the conditions of domain visibility and e-values<1.0. Domain searches in Pfam were performed with a cut-off e-value of 1.0. A protein was annotated with a domain or motif only when found by at least two search engines.

#### Signal peptide identification

Three Web services were used to identify signal peptides in *A. marginale* strain St. Maries: SignalP 3.0 [48], Phobius [49], and Philius [50]. All Web servers were used with default settings and accessed in June 2010. A protein was annotated as containing a signal peptide when predicted by at least two servers. Additionally, for Philius, the confidence level was >90%.

#### Sequence identity search

Sequence identity searches were performed using BLAST v.2.2.23+ with the following parameters: e-value 10, max_target_seqs 3, best_hit_overhang 0.1, and best_hit_score_edge 0.1. Two-way BLAST analysis was performed for 145 *L. pneumophilia* effector protein sequences and for the complete *A. marginale* proteome. The top three results were retained for each run. The E-value describes the number of hits one can “expect” by chance when searching a database of a particular size. The lower the E-value, or the closer it is to zero, the more “significant” the match is. In our case, however, given the phylogenetic distance between some of the species, we set the E-value high so a larger list with more low-scoring hits would be reported.

### Reporter assay

#### Plasmid construction

The pJB2581 vector was used for expression of CyaA fusion proteins in *L. pneumophila*. Full length *A. marginale* genes or the C-terminal 300 bp of each gene were amplified from genomic DNA by PCR using Accuprime *pfx* DNA polymerase (Invitrogen) and gene-specific primers (Integrated DNA Technologies, Coralville, IA) where the 5′ and 3′ primers contain a 16 bp linker complementary to the 5′ and 3′ ends of BamHI/SalI-digested pJB2581 (Table 2). Resulting PCR products were cloned into BamHI/SalI-digested pJB2581 using the In-Fusion Kit (Clontech). All plasmid inserts were sequenced to verify correct individual clones.

**Table 2:** Primer sequences used in this study.

Primer name	Sequence (5′ to 3′)
AM185-Full-F	TTCCGGCTATGGATCCATGTCGTCCGGAGGTGTG
AM185-c100aa-F	TTCCGGCTATGGATCCCAGCTTGTGAAAACCGATAATAC
AM185-R	ATGCCTGCAGGTCGACTTATCTACCACGCGCGCTTTG
AM410-Full-F	TTCCGGCTATGGATCCATGAGCACCGATAAAAATTTGCAGG
AM410-c100aa-F	TTCCGGCTATGGATCCGCAGCAGCAGTGGGGCCGTATG
AM410-R	ATGCCTGCAGGTCGACTCATGCATCTCTGCCGGTTG
AM470-Full-F	TTCCGGCTATGGATCCATGAAAAAGAAAACTAAACAATCGAC
AM470-c100aa-F	TTCCGGCTATGGATCCCGGCAATCAACGCAGCAGG
AM470-R	ATGCCTGCAGGTCGACTTAACGCGCAGCTCCTC
AM638-c100aa-F*	TTCCGGCTATGGATCCGCCAAACAAACGCTGGAAAG
AM638-R	ATGCCTGCAGGTCGACCTATTGCCGCTCCATTCTC
AM705-Full-F	TTCCGGCTATGGATCCATGTCTGAAGAATCTCTCATGGC
AM705-c100aa-F	TTCCGGCTATGGATCCGAACAAAAAACAAAGGCCAC
AM705-R	ATGCCTGCAGGTCGACTTACCAGCCTCTGGACAGG
AM878-Full-F	TTCCGGCTATGGATCCGTGATTGTGACATATGGCACTGTG
AM878-c100aa-F	TTCCGGCTATGGATCCGCGATTGAAGATGGCTTTG
AM878-R	ATGCCTGCAGGTCGACCTAGGACCCCAAGCATCCAAG
AM926-Full-F	TTCCGGCTATGGATCCATGTGTGGTGTGAGGAGGGTTTTTTTGACC
AM926-c100aa-F	TTCCGGCTATGGATCCAAGGCCAATGACATCACAGG
AM926-R	ATGCCTGCAGGTCGACCTACCCCTCTTGTTCTTCTTCGC
AM1141-Full-F	TTCCGGCTATGGATCCGTGCCGGCTTGTACTGAGC
AM1141-c100aa-F	TTCCGGCTATGGATCCGGGCGCGGCGAGACCAAGCTC
AM1141-R	ATGCCTGCAGGTCGACCTACCCTCTTCTGTGCGCAG

*The full length of AM638 was not cloned because of its large size.

#### *L. pneumophila* growth and transformation

*L. pneumophila* JR32 (wild type) and LELA3118 (DotA-deficient) strains [51] were cultured on charcoal yeast extract (CYE) agar plates. *L. pneumophila* transformations were conducted as previously described [7]. For plasmid selection, CYE plates contained 10 µg/ml chloramphenicol. For culture of *L. pneumophila* LELA3118, plates also contained 25 µg/ml kanamycin.

#### *L. pneumophila* CyaA translocation assays

*L. pneumophila* transformant cultures were incubated with 1 mM IPTG (ICN Biomedicals, Costa Mesa, CA) for 2 h to induce fusion protein expression. Cultures were analyzed by sodium dodecyl sulfate-polyacrylamide gel electrophoresis (SDS-PAGE) and immunoblotting (Fig. S1) using a mouse monoclonal antibody directed against CyaA (clone 3D1; Santa Cruz Biotechnology, Santa Cruz, CA) to confirm fusion protein expression. Reacting proteins were detected using an anti-mouse IgG secondary antibody conjugated to horseradish peroxidase (Pierce, Rockford, IL) and chemiluminescence using ECL Pico reagent (Pierce). *L. pneumophila* CyaA assays were performed in differentiated THP-1 cells as previously described using the cAMP Enzymeimmunoassay (GE Healthcare, Piscataway, NJ) [7]. Positive secretion of CyaA-effector fusion proteins was scored as ≥2.5-fold more cytosolic cAMP than that for cells infected with organisms expressing CyaA alone. Confirmation of Dot/Icm-dependent secretion was conducted by repeating the assay with the *L. pneumophila* DotA^−^ mutant.

## Supporting Information

Figure S1 **Verification of candidate protein expression.** Western blot analysis of *L. pneumophila* lysates from each expression construct probed with anti-CyaA monoclonal antibody.
(DOC)

Table S1 ***A. marginale*** **str. St. Maries 20 housekeeping genes.**
(DOC)

Table S2 ***A. tumefaciens*** **effectors.**
(DOC)

Table S3 ***B. henselae*** **effectors.**
(DOC)

Table S4 ***L. pneumophila*** **effectors.**
(DOC)
