# Supplementary material for: Identification of Anaplasma marginale Type IV Secretion System Effector Proteins
Source: PLoS One. 2011 Nov 28;6(11):e27724. doi: 10.1371/journal.pone.0027724 (PMC3225360; doi:10.1371/journal.pone.0027724)
Supplement: Figure S1 — Verification of candidate protein expression. Western blot analysis of L. pneumophila lysates from each expression construct probed with anti-CyaA monoclonal antibody. (DOC) [file pone.0027724.s001.doc]

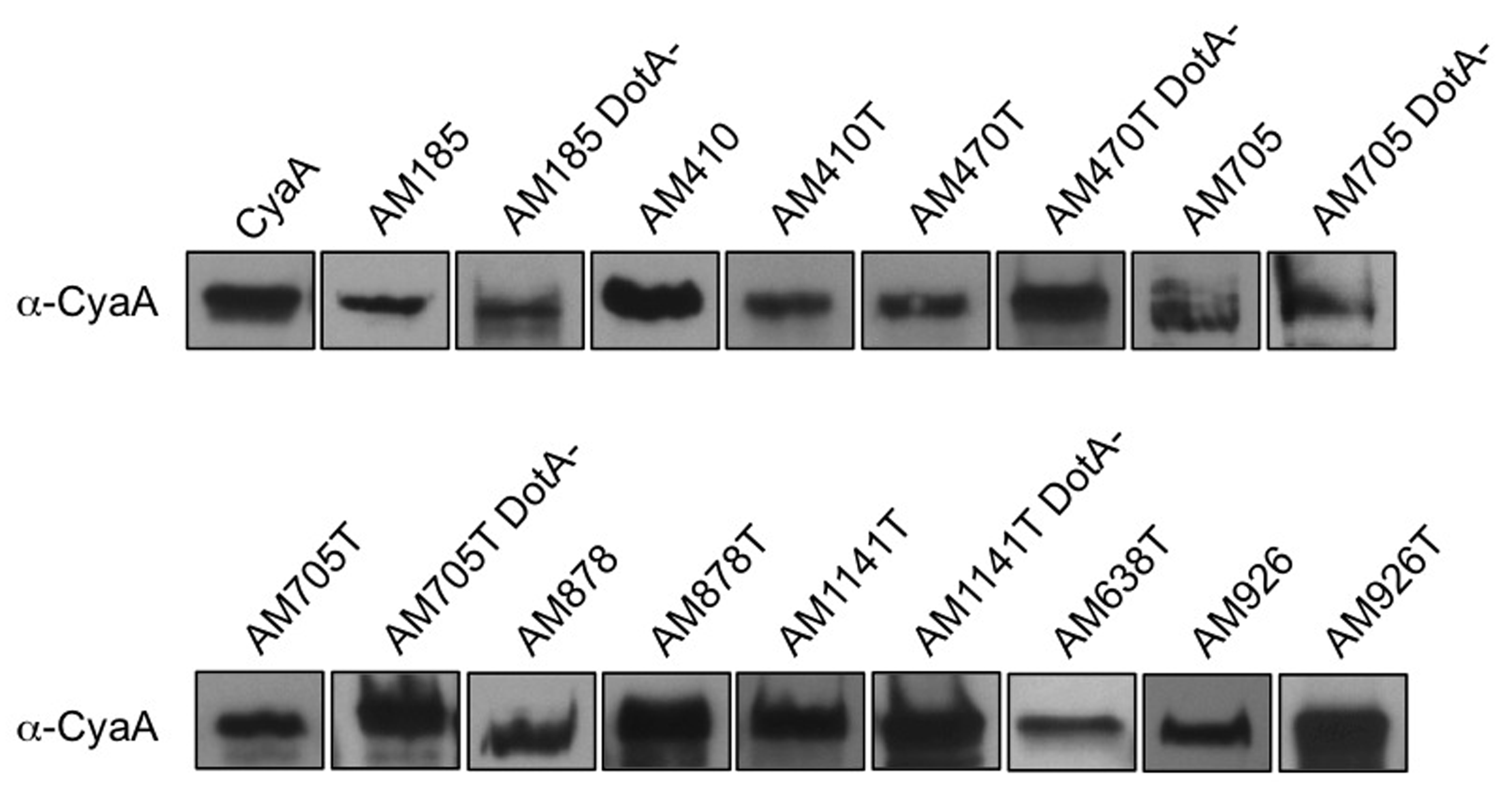


Figure S1. Verification of candidate protein expression. Western blot analysis of *L. pneumophila* lysates from each expression construct probed with anti-CyaA monoclonal antibody.
