# Supplementary material for: Identification of Anaplasma marginale Type IV Secretion System Effector Proteins
Source: PLoS One. 2011 Nov 28;6(11):e27724. doi: 10.1371/journal.pone.0027724 (PMC3225360; doi:10.1371/journal.pone.0027724)
Supplement: Table S1 — A. marginale str. St. Maries 20 housekeeping genes. (DOC) [file pone.0027724.s002.doc]

Table S1. *A. marginale* str. St. Maries 20 housekeeping genes^§§^.

| **Locus ID** | **Gene name /**  **GenBank ID** | **Length^1^** | **Hydro^2^** | **C-term charge^3^** | **C-term hydro^4^** | **Avg. hydro^5^** | **Protein Description** |
| --- | --- | --- | --- | --- | --- | --- | --- |
| AM130 | frr/YP_153529.1 | 183 | -13.90 | +0 | 0.7 | -0.08 | ribosome recycling factor |
| AM1274 | infC/YP_154330.1 | 177 | -88.70 | +1 | -7.8 | -0.50 | translation initiation factor IF-3 |
| AM1311 | infC/YP_154358.1 | 85 | -25.90 | +1 | -7.8 | -0.30 | translation initiation factor IF-3 |
| AM736 | nusA/YP_153941.1 | 515 | -77.30 | +3 | -9 | -0.15 | transcription elongation factor |
| AM1326 | pgk/YP_154370.1 | 385 | 54.80 | -2 | 11.4 | 0.14 | phosphoglycerate kinase |
| AM018 | pyrG/YP_153458.1 | 560 | 0.30 | +4 | 14.3 | 0.00 | CTP synthetase |
| AM259 | rplA/YP_153617.1 | 224 | 4.10 | +4 | 2.9 | 0.02 | 50S ribosomal protein |
| AM909 | rplB/YP_154074.1 | 277 | -82.00 | +12 | -48.9 | -0.30 | 50S ribosomal protein |
| AM912 | rplC/YP_154077.1 | 232 | -6.00 | +2 | -7 | -0.03 | 50S ribosomal protein |
| AM911 | rplD/YP_154076.1 | 208 | -17.80 | +1 | 11.2 | -0.09 | 50S ribosomal protein |
| AM899 | rplE/YP_154065.1 | 179 | 20.70 | -1 | 6.2 | 0.12 | 50S ribosomal protein |
| AM896 | rplF/YP_154062.1 | 181 | -14.20 | +5 | -31.2 | -0.08 | 50S ribosomal protein |
| AM258 | rplK/YP_153616.1 | 147 | 4.70 | -2 | 5.5 | 0.03 | 50S ribosomal protein |
| AM261 | rplL/YP_153619.1 | 134 | -32.70 | +3 | -28 | -0.24 | 50S ribosomal protein |
| AM1023 | rplM/YP_154159.1 | 170 | -57.30 | +2 | -25.6 | -0.34 | 50S ribosomal protein |
| AM901 | rplN/YP_154067.1 | 119 | 28.00 | +2 | 16.6 | 0.24 | 50S ribosomal protein |
| AM905 | rplP/YP_154070.2 | 138 | -34.60 | +3 | -9.9 | -0.25 | 50S ribosomal protein |
| AM1271 | rplS/YP_154327.1 | 134 | -53.30 | +6 | -28.6 | -0.40 | 50S ribosomal protein |
| AM054 | rplT/YP_153481.1 | 128 | -56.70 | 0 | 6.5 | -0.44 | 50S ribosomal protein |
| AM596 | rpmA/YP_153857.1 | 90 | -32.60 | +3 | 14.5 | -0.36 | 50S ribosomal protein |
| AM262 | rpoB/YP_153620.1 | 1382 | -150.30 | -1 | -5.8 | -0.11 | DNA polymeraze |
| AM792 | rpsB/YP_153979.2 | 327 | -52.00 | -7 | -6.4 | -0.16 | 30S ribosomal protein |
| AM906 | rpsC/YP_154071.1 | 211 | -24.90 | 0 | 1.3 | -0.12 | 30S ribosomal protein |
| AM894 | rpsE/YP_154060.1 | 174 | -4.30 | +5 | -23.2 | -0.02 | 30S ribosomal protein |
| AM1022 | rpsI/YP_154158.1 | 153 | -42.60 | +10 | -50.9 | -0.28 | 30S ribosomal protein |
| AM913 | rpsJ/YP_154078.1 | 105 | 2.10 | +1 | 25.3 | 0.02 | 30S ribosomal protein |
| AM888 | rpsK/YP_154055.2 | 124 | -17.20 | +7 | -23.1 | -0.14 | 30S ribosomal protein |
| AM890 | rpsM/YP_154056.1 | 123 | -4.90 | +9 | -31.2 | -0.04 | 30S ribosomal protein |
| AM908 | rpsS/YP_154073.1 | 93 | -24.80 | +7 | -31.1 | -0.27 | 30S ribosomal protein |
| AM1171 | smpB/YP_154258.1 | 149 | -79.00 | +4 | -45.4 | -0.53 | SsrA-binding protein |
| AM791 | tsf/YP_153978.1 | 291 | 6.00 | +1 | 2.5 | 0.02 | elongation factor Ts |

^1^ Protein length in amino acids.

^2^ Hydropathy of total protein.

^3^ Charge of C-terminal 25 amino acids.

^4^ Hydropathy of C-terminal 25 amino acids.

^5^ Average hydropathy = total hydropathy / length.

**^§§^** Reference to the list of 31 housekeeping genes is from *Methods* in “**Comparative Genomics of Emerging Human Ehrlichiosis Agents**” by Dunning Hotopp JC, Lin M, Madupu R, Crabtree J, Angiuoli SV, et al. (2006), *PLoS Genet* 2(2): e21.
