# Supplementary material for: Identification of Anaplasma marginale Type IV Secretion System Effector Proteins
Source: PLoS One. 2011 Nov 28;6(11):e27724. doi: 10.1371/journal.pone.0027724 (PMC3225360; doi:10.1371/journal.pone.0027724)
Supplement: Table S2 — A. tumefaciens effectors. (DOC) [file pone.0027724.s003.doc]

Table S2. *A. tumefaciens* effectors.

| ***Protein Name/ UniProtKB ID*** | **Length^1^** | **Hydro^2^** | **C-term charge^3^** | **C-term hydro^4^** | **Avg. hydro^5^** |
| --- | --- | --- | --- | --- | --- |
| VirD2/Q79AU0 | 424 | -419.7 | +4 | -57.5 | -0.99 |
| VirD5/Q9JN11 | 833 | -658.9 | +1 | -44.4 | -0.79 |
| VirE2/P0A3W8 | 533 | -446.4 | +3 | -30.4 | -0.84 |
| VirE3/Q44445 | 672 | -594.1 | +5 | -33.8 | -0.88 |
| VirF/Q79AT3 | 202 | -112 | +2 | -23.7 | -0.55 |

^1^ Protein length in amino acids.

^2^ Hydropathy of total protein.

^3^ Charge of C-terminal 25 amino acids.

^4^ Hydropathy of C-terminal 25 amino acids.

^5^ Average hydropathy = total hydropathy / length.
