# Supplementary material for: Identification of Anaplasma marginale Type IV Secretion System Effector Proteins
Source: PLoS One. 2011 Nov 28;6(11):e27724. doi: 10.1371/journal.pone.0027724 (PMC3225360; doi:10.1371/journal.pone.0027724)
Supplement: Table S3 — B. henselae effectors. (DOC) [file pone.0027724.s004.doc]

Table S3. *B. henselae* effectors.

| ***Protein Name/ UniProtKB ID*** | **Length^1^** | **Hydro^2^** | **C-term charge^3^** | **C-term hydro^4^** | **Avg. hydro^5^** |
| --- | --- | --- | --- | --- | --- |
| BepA/Q6G2A9 | 544 | -265.1 | +5 | -21.8 | -0.49 |
| BepB/Q6G2A7 | 542 | -299.3 | +5 | -14.2 | -0.55 |
| BepC/Q6G2A6 | 532 | -342.3 | +5 | -16.1 | -0.64 |
| BepD/Q6G2A5 | 534 | -625.5 | +1 | -41.2 | -1.17 |
| BepE/Q6G2A4 | 464 | -459.4 | +5 | -14.1 | -0.99 |
| BepF/Q6G2A3 | 834 | -936.3 | +3 | -26.6 | -1.12 |
| BepG/Q5QSZ9 | 1009 | -513.9 | +3 | -3.4 | -0.51 |

^1^ Protein length in amino acids.

^2^ Hydropathy of total protein.

^3^ Charge of C-terminal 25 amino acids.

^4^ Hydropathy of C-terminal 25 amino acids.

^5^ Average hydropathy = total hydropathy / length.
