# Supplementary material for: Identification of Anaplasma marginale Type IV Secretion System Effector Proteins
Source: PLoS One. 2011 Nov 28;6(11):e27724. doi: 10.1371/journal.pone.0027724 (PMC3225360; doi:10.1371/journal.pone.0027724)
Supplement: Table S4 — L. pneumophila effectors. (DOC) [file pone.0027724.s005.doc]

Table S4. *L. pneumophila* effectors.

| **Gene ID** | **Length^1^** | **Hydro^2^** | **C-term charge^3^** | **C-term hydro^4^** | **Avg. hydro^5^** | **A.marginale locus tag**^6^ |
| --- | --- | --- | --- | --- | --- | --- |
| lpg0012 | 525 | -254.2 | 0 | -7.8 | -0.48 |  |
| lpg0038 | 507 | -77.2 | -1 | -23.6 | -0.15 | AM705 |
| lpg0045 | 70 | -101 | +4 | -29.6 | -1.44 |  |
| lpg0080 | 255 | -105.8 | -1 | -12.8 | -0.41 |  |
| lpg0081 | 441 | -126.1 | +2 | -17.2 | -0.29 |  |
| lpg0090 | 1324 | -395.4 | -2 | -11.6 | -0.30 |  |
| lpg0096 | 397 | -176.8 | +1 | -41.5 | -0.45 |  |
| lpg0103 | 286 | -62.3 | +1 | 1.7 | -0.22 |  |
| lpg0126 | 1102 | -281 | -1 | 2.7 | -0.25 |  |
| lpg0135 | 1875 | -717.7 | -1 | -29.4 | -0.38 |  |
| lpg0171 | 188 | -28.3 | -4 | -23.6 | -0.15 |  |
| lpg0191 | 317 | -95 | -2 | -7.4 | -0.30 | AM712 |
| lpg0227 | 368 | -180.3 | -3 | -7.7 | -0.49 |  |
| lpg0234 | 1514 | -809 | +1 | -19.6 | -0.53 |  |
| lpg0240 | 259 | -81.2 | -1 | -5 | -0.31 |  |
| lpg0246 | 241 | -71.9 | -1 | -17 | -0.30 |  |
| lpg0275 | 1116 | -478.3 | -1 | -11.2 | -0.43 |  |
| lpg0276 | 499 | -286.6 | -7 | -5 | -0.57 |  |
| lpg0284 | 374 | -267 | 0 | -9.9 | -0.71 |  |
| lpg0285 | 227 | 11.4 | -1 | -6.8 | 0.05 |  |
| lpg0294 | 230 | -124.6 | -2 | -30.2 | -0.54 |  |
| lpg0365 | 896 | -182.7 | +1 | -15.3 | -0.20 |  |
| lpg0376 | 1429 | -703.6 | -1 | -3.3 | -0.49 |  |
| lpg0390 | 352 | -202.7 | +4 | -52 | -0.58 |  |
| lpg0402 | 567 | -55.5 | +1 | -19.1 | -0.10 |  |
| lpg0403 | 514 | -191.1 | +5 | -22.5 | -0.37 | AM638 |
| lpg0436 | 269 | -125.3 | -1 | -23.5 | -0.47 |  |
| lpg0437 | 666 | -302.3 | 0 | -44.8 | -0.45 |  |
| lpg0483 | 495 | -147.3 | 0 | -17.7 | -0.30 |  |
| lpg0518 | 282 | 53.9 | -2 | -18.6 | 0.19 |  |
| lpg0519 | 737 | -219.3 | +1 | 18 | -0.30 |  |
| lpg0621 | 474 | -23.9 | -1 | -33.2 | -0.05 |  |
| lpg0634 | 449 | -198 | -8 | -44.2 | -0.44 |  |
| lpg0642 | 524 | -211.1 | +2 | -13.7 | -0.40 |  |
| lpg0695 | 949 | -355.2 | +3 | -22.6 | -0.37 | AM705 |
| lpg0696 | 570 | -229.4 | +1 | -20.2 | -0.40 |  |
| lpg0898 | 243 | -42.9 | -2 | -16.6 | -0.18 |  |
| lpg0940 | 729 | -674 | +1 | -22.5 | -0.92 |  |
| lpg0945 | 296 | -44.8 | 0 | -18.2 | -0.15 | AM071 |
| lpg0963 | 413 | -5.5 | -2 | -14.1 | -0.01 |  |
| lpg1101 | 322 | -205.9 | +1 | -15.5 | -0.64 | AM712 |
| lpg1110 | 231 | -42.8 | +1 | -4 | -0.19 |  |
| lpg1120 | 588 | -338.8 | -1 | -33.2 | -0.58 |  |
| lpg1121 | 256 | -72.4 | -3 | -3.2 | -0.28 |  |
| lpg1144 | 168 | -123.5 | -8 | -46.6 | -0.74 |  |
| lpg1145 | 769 | -219.2 | -1 | -16.2 | -0.29 |  |
| lpg1148 | 503 | -192.4 | -2 | -21.4 | -0.38 |  |
| lpg1158 | 256 | -134.6 | -2 | -27.2 | -0.53 |  |
| lpg1227 | 598 | -239.2 | -1 | -27.7 | -0.40 |  |
| lpg1273 | 354 | -145.2 | -4 | 2.2 | -0.41 |  |
| lpg1290 | 528 | -373.2 | 0 | -55.1 | -0.71 | AM638 |
| lpg1355 | 974 | -638.6 | -6 | -28.4 | -0.66 |  |
| lpg1426 | 884 | -359 | +3 | -21.8 | -0.41 | AM712 |
| lpg1483 | 529 | -219.8 | 0 | 0.5 | -0.42 |  |
| lpg1488 | 865 | -506.4 | 0 | -14.8 | -0.59 |  |
| lpg1491 | 413 | -216.7 | 0 | -23.1 | -0.52 |  |
| lpg1496 | 598 | -234.4 | -1 | -19.8 | -0.39 |  |
| lpg1588 | 672 | -330.1 | -1 | -18.5 | -0.49 |  |
| lpg1598 | 353 | -100.1 | +1 | -12.4 | -0.28 |  |
| lpg1602 | 428 | 22.3 | +1 | -3.6 | 0.05 |  |
| lpg1621 | 439 | -125.2 | +2 | -20.1 | -0.29 |  |
| lpg1625 | 130 | -56.2 | +2 | 32.4 | -0.43 |  |
| lpg1642 | 417 | -168.4 | 0 | -13.2 | -0.40 |  |
| lpg1660 | 493 | -107.5 | +3 | -30.8 | -0.22 |  |
| lpg1689 | 208 | -9.1 | -2 | -11.8 | -0.04 |  |
| lpg1701 | 560 | -270.8 | -9 | -25.3 | -0.48 | AM705 |
| lpg1702 | 546 | -278.4 | +2 | -2.9 | -0.51 |  |
| lpg1717 | 562 | -215.1 | +1 | 13.5 | -0.38 |  |
| lpg1718 | 545 | -254.1 | -3 | 6.1 | -0.47 |  |
| lpg1751 | 436 | -139.9 | +1 | -18.5 | -0.32 |  |
| lpg1836 | 472 | -368 | 0 | -53.6 | -0.78 |  |
| lpg1851 | 220 | -136.5 | +2 | -17.2 | -0.62 |  |
| lpg1884 | 405 | -212 | -4 | -23.3 | -0.52 | AM071 |
| lpg1890 | 573 | -231.8 | -1 | -23.5 | -0.40 |  |
| lpg1933 | 204 | 30.9 | +1 | -19.6 | 0.15 |  |
| lpg1947 | 249 | -190.5 | 0 | -1.7 | -0.77 | AM387 |
| lpg1948 | 335 | -56.4 | +1 | 6.4 | -0.17 |  |
| lpg1949 | 446 | -327.5 | 0 | -32.4 | -0.73 |  |
| lpg1950 | 374 | -211.7 | +1 | -28.6 | -0.57 |  |
| lpg1953 | 760 | -280.4 | -3 | 3.2 | -0.37 |  |
| lpg1958 | 542 | -45.3 | +1 | -8.2 | -0.08 |  |
| lpg1960 | 257 | -188.5 | +1 | -43.1 | -0.73 |  |
| lpg1962 | 188 | -12.3 | 0 | -9.2 | -0.07 |  |
| lpg1963 | 699 | -489.3 | +1 | -22.5 | -0.70 |  |
| lpg1964 | 436 | -177.6 | -4 | -20 | -0.41 |  |
| lpg1965 | 988 | -484.2 | +1 | -28.2 | -0.49 |  |
| lpg1966 | 521 | -228.8 | -1 | -20.9 | -0.44 |  |
| lpg1969 | 635 | -463.2 | -3 | -19.5 | -0.73 |  |
| lpg1976 | 286 | -87.4 | +3 | -8.7 | -0.31 |  |
| lpg1978 | 644 | -318.3 | +1 | -8.4 | -0.49 |  |
| lpg2137 | 538 | -264.8 | +1 | -21.9 | -0.49 | AM185 |
| lpg2144 | 172 | -107.7 | +3 | -11 | -0.63 |  |
| lpg2153 | 1533 | -885.1 | -4 | -32.4 | -0.58 |  |
| lpg2154 | 301 | -126.9 | -1 | -26.1 | -0.42 |  |
| lpg2155 | 873 | -478.2 | 0 | -51.2 | -0.55 |  |
| lpg2156 | 1926 | -1193.2 | -2 | -37.1 | -0.62 |  |
| lpg2157 | 1506 | -845.9 | -1 | -19.1 | -0.56 |  |
| lpg2166 | 419 | -297.4 | 0 | 15.2 | -0.71 |  |
| lpg2176 | 608 | -126.2 | +1 | -26.9 | -0.21 |  |
| lpg2200 | 178 | -183.4 | 0 | -27.3 | -1.03 |  |
| lpg2215 | 531 | -155.8 | +4 | 2.6 | -0.29 |  |
| lpg2216 | 590 | -404.9 | -1 | 9.6 | -0.69 |  |
| lpg2248 | 744 | -433.1 | +3 | 0.9 | -0.58 |  |
| lpg2298 | 425 | -192.3 | +1 | -12.6 | -0.45 |  |
| lpg2300 | 467 | -79.7 | -1 | 7.9 | -0.17 |  |
| lpg2322 | 641 | -304.3 | -1 | -4.8 | -0.47 | AM638 |
| lpg2327 | 297 | -256.8 | 0 | -26.9 | -0.86 | AM712 |
| lpg2328 | 127 | -93.6 | -2 | -21.3 | -0.74 |  |
| lpg2391 | 434 | -172.1 | -2 | -22.8 | -0.40 |  |
| lpg2400 | 354 | 23.3 | -1 | -25 | 0.07 |  |
| lpg2406 | 365 | -213.8 | +1 | -16.2 | -0.59 |  |
| lpg2407 | 101 | -76.6 | -1 | -22 | -0.76 |  |
| lpg2409 | 272 | -173.2 | +2 | -40.9 | -0.64 |  |
| lpg2410 | 665 | -191.9 | 0 | -17.4 | -0.29 |  |
| lpg2411 | 275 | -152.2 | -1 | -18.6 | -0.55 | AM638 |
| lpg2422 | 753 | -615.1 | 0 | -5.5 | -0.82 |  |
| lpg2433 | 586 | -200.9 | +3 | -5.8 | -0.34 |  |
| lpg2452 | 921 | -367.7 | -2 | -19.7 | -0.40 |  |
| lpg2456 | 471 | -308.7 | +1 | -15.8 | -0.66 |  |
| lpg2464 | 647 | -380.1 | +1 | -31.3 | -0.59 |  |
| lpg2465 | 507 | -81.3 | +1 | -1.6 | -0.16 |  |
| lpg2482 | 448 | -292.7 | -1 | -25.2 | -0.65 |  |
| lpg2490 | 1294 | -849 | +1 | -12.6 | -0.66 |  |
| lpg2504 | 942 | -607.6 | +1 | -17.3 | -0.65 | AM387 |
| lpg2508 | 807 | -336.4 | +1 | -40.9 | -0.42 |  |
| lpg2509 | 397 | -175.7 | +3 | -30.6 | -0.44 |  |
| lpg2510 | 908 | -685.5 | +2 | -37.3 | -0.75 | AM712 |
| lpg2511 | 917 | -684.4 | 0 | -21.8 | -0.75 |  |
| lpg2523 | 779 | -426.8 | -1 | -26.9 | -0.55 |  |
| lpg2527 | 558 | -397 | -2 | -39.9 | -0.71 |  |
| lpg2529 | 572 | -326.4 | 0 | -19.5 | -0.57 |  |
| lpg2556 | 462 | -260.9 | -2 | -8.5 | -0.56 |  |
| lpg2584 | 912 | -626.7 | -3 | -20.8 | -0.69 |  |
| lpg2591 | 165 | -52 | 0 | -23 | -0.32 |  |
| lpg2603 | 434 | -326.7 | +2 | -6.6 | -0.75 |  |
| lpg2718 | 520 | -197.6 | +2 | -8.6 | -0.38 |  |
| lpg2744 | 389 | -152.2 | -1 | -10 | -0.39 |  |
| lpg2793 | 1151 | -636.7 | +2 | -7.3 | -0.55 |  |
| lpg2804 | 468 | 6.9 | -1 | -22.8 | 0.01 |  |
| lpg2826 | 577 | -356.8 | -4 | -28.2 | -0.62 |  |
| lpg2829 | 2225 | -1210.4 | -3 | -12.3 | -0.54 |  |
| lpg2830 | 246 | -128.6 | -1 | -22.6 | -0.52 |  |
| lpg2831 | 621 | -259 | +1 | -31.1 | -0.42 |  |
| lpg2862 | 636 | -243.2 | 0 | -26.6 | -0.38 |  |
| lpg2999 | 266 | -106.2 | 0 | -10.4 | -0.40 |  |
| **Median** |  | -211.1 |  |  |  |  |
| **Average** |  | -261.2 |  |  |  |  |

^1^ Protein length in amino acids.

^2^ Hydropathy of total protein.

^3^ Charge of C-terminal 25 amino acids.

^4^ Hydropathy of C-terminal 25 amino acids.

^5^ Average hydropathy = total hydropathy / length.

^6^ Protein of *A. marginale St. Maries* that has some sequence identity with corresponding *L. pneumophila* protein (see Table 1 in main text).
